# Thermal mechanism in magneto radiated [(Al_2_O_3_-Fe_3_O_4_)/blood]_hnf_ over a 3D surface: Applications in Biomedical Engineering

**DOI:** 10.3389/fchem.2022.960349

**Published:** 2022-10-06

**Authors:** Kamel Guedri, Adnan, Zehba Raizah, Elsayed Tag Eldin, M. A. EL-Shorbagy, Waseem Abbas, Umar Khan

**Affiliations:** ^1^ Mechanical Engineering Department, College of Engineering and Islamic Architecture, Umm Al-Qura University, Makkah, Saudi Arabia; ^2^ Department of Mathematics, Mohi-ud-Din Islamic University, Nerian Sharif, AJ&K, Pakistan; ^3^ Department of Mathematics, College of Science, King Khalid University, Abha, Saudi Arabia; ^4^ Research Center for Advanced Materials Science (RCAMS), King Khalid University, Abha, Saudi Arabia; ^5^ Faculty of Engineering and Technology, Future University in Egypt New Cairo, New Cairo, Egypt; ^6^ Department of Mathematics, College of Science and Humanities in Al-Kharj, Prince Sattam Bin Abdulaziz University, Al-Kharj, Saudi Arabia; ^7^ Department of Basic Engineering Science, Faculty of Engineering, Menoufia University, Shebin El-Kom, Egypt; ^8^ Department of Mathematics and Statistics, Hazara University, Mansehra, Pakistan

**Keywords:** thermal enhancement, Al2O3-Fe3O4 hybrid nanoparticles, blood, thermal radiation, slip boundaries

## Abstract

Nanofluids are a new generation of fluids which help in improving the efficiency of thermal systems by improving heat transport rate and extensive applications of this class extensively fall in biomedical engineering, the electronics industry, applied thermal and mechanical engineering, etc. The core concern of this study is to examine the interaction of Al_2_O_3_-Fe_3_O_4_ hybrid nanoparticles of lamina shaped with blood over a 3D surface by impinging novel impacts of non-linear thermal radiations, stretching, velocity slippage, and magnetic field. This leads to a mathematical flow model in terms of highly non-linear differential equations *via* nanofluid-effective characteristics and similarity rules. To know the actual behavior of (Al_2_O_3_-Fe_3_O_4_)/blood inside the concerned region, mathematical investigation is performed *via* numerical technique and the results are obtained for different parameter ranges. The imposed magnetic field of high strength is a better tool to control the motion of (Al_2_O_3_-Fe_3_O_4_)/blood inside the boundary layer, whereas, stretching of the surface is in direct proportion of the fluid movement. Furthermore, thermal radiations (Rd) and $\gamma_{1}$ are observed to be beneficial for thermal enhancement for both (Al_2_O_3_-Fe_3_O_4_)/blood and (Al_2_O_3_)/blood.

## Introduction

The world of nanotechnology is nothing without the investigation of the dynamics of nano and hybrid fluids (Alharbi et al., 2022). Now-a-days, we see that many researchers have come out with new technological ideas of a hybrid nanofluid, which is an upgraded version of common liquids. Hybrid nanofluids have high thermal conductivity due to the joint contribution of two types of nanoparticles. Therefore, researchers have seriously taken the analysis of such fluids from synthetization to applications and have performed the studies at a high level. Recently, Kashi et al. (Kashi et al., 2020) demonstrated the study of (Cu-Al_2_O_3_/water) in three dimensions over a slippage surface with uniform surface convection. Another imperative study related to the thermal behavior of Cu-Al_2_O_3_/water by taking flow assumptions on the surface was conveyed in (Khashi et al., 2020).

The transmission of heat in a hybrid nanoliquid with water as the base component and (Al_2_O_3_-Cu) hybrid nanoparticles was examined by Zainal et al. (Zaina et al., 2020). The inspection of Lorentz forces in the flow behavior over a 3D surface subject to resistive heating is done in Devi and Devi, 2016a. Hybrid nanofluids are a new generation of heat transport fluids with enriched energy storage ability. Therefore, Devi et al. (Devi and Devi, 2016b) reported a comparative heat transport performance of two nanofluids over a permeable surface. Recently, the stability analysis of (Cu-Al_2_O_3_/water) (Khan et al., 2022) over a non-linear shrinkable sheet and the heat dynamics under certain physical constraints is described in (Lund et al., 2019). Usman et al. (Usman et al., 2018) studied the significant effects of non-linear thermal radiations (Khan et al., 2021) with the contribution of thermal conductance on the temperature of nanoliquids and explored that the imposition of thermal radiation as an enriched natural source to boost the heat storage ability of the nanoliquids.

The investigation of momentum slippage and MHD (Ahmed, Adnan, and Mohyud-Din, 2020) on Cu-Al_2_O_3_/water nanofluid flow over a permeable stretching sheet is described in Wahid et al., 2020. Furthermore, a study of nanoliquids influenced by gravity is examined in Jamaludin et al., 2020. A hybrid nanofluid with surface temperature and Lorentz forces was examined by Prakash et al.,2016 and concluded that the hybrid nanofluid had better efficiency than traditional nanofluids. Another significant contribution in thermal enhancement is reported in Colak et al., 2020 to estimate the specific heat of Cu-Al_2_O_3_/water hybrid nanofluid based on temperature (T) and volume concentrations (*φ*). Mehryan et al. (Mehryan et al., 2017) studied the free convection thermal performance in a cavity with nanofluids and pointed out that the thermophysical attributes of the nanoparticles empower the thermal transport rate in nanoliquids.

Lund et al. (Lund et al., 2020) explored the thermal characteristics of Cu-Al_2_O_3_/water by considering MHD and viscous dissipation insights over a shrinkable sheet. Alshare et al. (Alshare et al., 2020) investigated the nano and hybrid nanofluid heat transport mechanisms in a periodic structure and found that enhancing % volume fraction will result increment in the temperature and frictional effects. A numerical analysis of an unsteady MHD mixed convection (Khan, Adnan, and Haleema, 2022) stagnation point flow heat transmission model (SPFM) for Cu-Al_2_O_3_/water over a 3D oriented geometry is described in Zainal et al., 2021. Nur et al. (Wahid et al., 2020) examined an analytic solution under slip momentum and thermal radiation influences on magnetized Cu-Al_2_O_3_/water nanofluid over a permeable stretching sheet (Adnan et al., 2022a). Force convection of turbulent flow of pure water, Al_2_O_3_/water nanofluid, and Cu-Al_2_O_3_/water hybrid nanofluid through a uniformly heated circular geometry is numerically analyzed in Takabi and Shokouhmand, 2015. Some other beneficial heat transport investigations in nanoliquids are reported in (Roy et al., 2020), (Adnan and Ahmed, 2022), (Leong et al., 2020), (Waqas et al., 2021), (Adnan and Ashraf, 2022a).

Jamshed et al. (Jamshad et al., 2021) studied the Casson nanofluid and examined the results for entropy and heat transport under solar thermal radiations. Sajid et al. (Sajid et al., 2021) studied the second law for a parabolic trough surface collector (PTSC) located inside solar aircraft wings, by taking the homo/heterogeneous reaction. Kashi et al. (Khashi et al., 2021) formulated the model for Cu-Al_2_O_3_/water hybrid nanofluid using the single-phase technique and reported a detailed analysis. Recent investigations were revealed in the studies by Adnan et al., 2020a, Adnan et al., 2022b, Adnan et al., 2020b, and Ahmed et al., 2017.

The study of electroosmotic silver/water nanoliquids in peristaltic geometry *via* two distinct approaches is done by Akram et al. (Akram et al., 2022a). They treated the developed model through different approaches and analyzed the dynamics due to fluctuating peristalsis parameters. An experimental analysis regarding the resistance of anti-microbes for gold nanoparticles is described by Habib and Akbar (Habib and Akbar, 2021) and the results are explained in a comprehensive manner. The exploration of an exact solution for various fluid dynamic heat transmission models is of great significance to examine the behavior of the temperature inside the concerned region. In this regard, a significant analysis is reported by Akbar et al. (Akbar et al., 2022). The impacts of thermal radiations on the nanoliquid whose components are CNTs and water are explained in detail. The entropy investigation in a new Rabinowitsch nanoliquid due to peristaltic pumping is discussed by Akram et al. (Akram et al., 2022b). Some recent and well-contributed studies in the area of applied fluid mechanics from various physical aspects (thermal radiations, magnetic field, heat sink/source inside the fluid, viscous dissipation, joule heating, etc.) of flow and geometry are elaborated in Akram and Akbar, 2020; Akram et al., 2020; Butt et al., 2020 at various spans of time.

The dynamics of nanoliquid in a curved channel with a thermophoretic movement are disclosed by Akram et al. (Akram et al., 2022c). The authors determined that higher buoyancy forces strengthened the temperature and facilitated the fluid movement. The temperature due to thermal radiations, resistive heating (Abbasi et al., 2017), and dissipation function in a nanoliquid prepared by gold and blood were analyzed by Sridhar et al. (Sridhar et al., 2022). The magnetic field (Akram et al., 2022d) is an important perspective from an industrial view point and broadly applicable in a variety of industries. Therefore, the researchers made several attempts to inspect the temperature transmission and fluid movement under a variety of nanoliquids by taking the flow in different regimes. Such important studies are described in the Refs. (Akram et al., 2022e; Tripathi et al., 2022), (Saleem et al., 2021), (Javid et al., 2021), and (Tripathi et al., 2021).

## Model development

### Model statement and geometry

**FIGURE 1:**
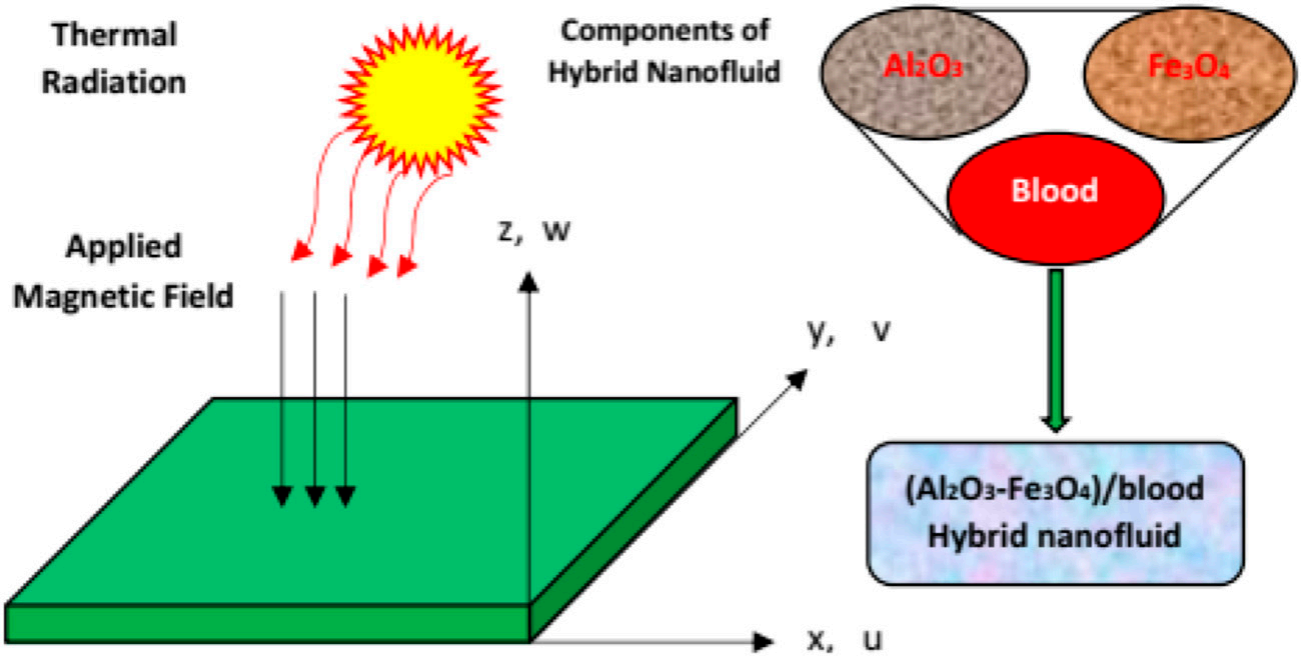
The Physical flow configuration of (Al_2_O_3_-Fe_3_O_4_)/blood and Al_2_O_3_/blood.

Consider a three-dimensional, steady, laminar flow over a surface with modified slip boundaries. The flow is incompressible and subject to the magnetic field. Furthermore, thermal radiations are also imposed over the surface for better thermal performance of the nanofluids. The *x* and *y* axes are designated along the length and width of the stretching sheet, respectively; while the *z*-direction is taken perpendicular to the sheet. We take u, v, and w, as the velocity components along the *x*, *y,* and *z* directions, respectively. Furthermore, we imposed a magnetic field **B**_**o**_ with uniform strength and aligned along *z*-axis. The flow region for the used nanofluids is depicted in Figure 1.

The steady Prandtl boundary layer flow of the nanoliquid can be described by the following PDEs (Devi and Devi, 2016a), (Hayat et al., 2015):

$$\frac{\partial \bar{u}}{\partial x}+\frac{\partial \bar{v}}{\partial y}+\frac{\partial \bar{w}}{\partial z}=0,$$ (1)

$$\bar{u}\frac{\partial \bar{u}}{\partial x}+\bar{v}\frac{\partial \bar{u}}{\partial y}+\bar{w}\frac{\partial \bar{u}}{\partial z}=V_{hnf}\frac{\partial^{2}\bar{u}}{\partial x^{2}}-\frac{\sigma_{hnf}B_{ο}^{2}\bar{u}}{\rho_{hnf}},$$ (2)

$$\bar{u}\frac{\partial \bar{v}}{\partial x}+\bar{v}\frac{\partial \bar{v}}{\partial y}+\bar{w}\frac{\partial \bar{u}}{\partial z}=V_{hnf}\frac{\partial^{2}\bar{v}}{\partial z^{2}}-\frac{\sigma_{hnf}B_{о}^{2}}{\rho_{hnf}}\bar{v},$$ (3)

$$\bar{u}\frac{\partial T}{\partial x}+\bar{v}\frac{\partial T}{\partial y}+\bar{w}\frac{\partial T}{\partial z}=\alpha_{hnf}\frac{\partial^{2}T}{\partial z^{2}}+\frac{16\sigma^{*}T_{\infty }^{3}}{3k^{*}\left(\rho Cp_{hnf}\right)}\frac{\partial^{2}T}{\partial z^{2}}.$$ (4)

The flow on the boundaries is specified to the following rules (Hayat et al., 2015):

$$\{\begin{array}{c} \bar{u}=ax+\frac{\left(2-\sigma_{\nu }\right)}{\sigma_{\nu }}\lambda_{0}\frac{\partial \bar{u}}{\partial z}\\ \bar{v}=by+\frac{\left(2-\sigma_{\nu }\right)}{\sigma_{\nu }}\lambda_{0}\frac{\partial \bar{v}}{\partial z}\\ \bar{u}\to 0,\;\bar{v}\to 0,\;T\to T_{\infty },\;as\;z\to \infty \end{array}\bar{w}=0,\}at\;z=0$$ (5)

The quantities appearing in the aforementioned governing laws are $V_{hnf}$ (Kinematic viscosity), $\rho_{hnf}$ (Density), $k_{hnf}$ (Thermal conductivity), $\alpha_{hnf}$ (Thermal diffusivity), $B_{O}$ (Uniform magnetic field), and a and b are constants representing the stretching surface rate.

### Thermo-physical attributes for nano and hybrid fluids

The following are thermophysical attributes of nano and hybrid fluids utilized to modify the problem for (Al_2_O_3_-Fe_3_O_4_)/blood and Al_2_O_3_/blood over a desired 3D stretchable surface (Khan et al., 2017; Adnan and Ashraf, 2022b):

$\rho_{nf}=\left(1-ф\right)\rho_{s}$ (density),

${\left(\rho C_{p}\right)}_{nf}=\left(1-ф\right){\left(\rho C_{p}\right)}_{f}+ф{\left(\rho C_{p}\right)}_{s}$ (heat capacity),

${µ}_{nf}=\frac{{µ}_{f}}{{\left(1-ф\right)}^{2.5}}$ (dynamic viscosity),

$\frac{k_{nf}}{k_{f}}=\left(\frac{k_{\text{s}1}+\left(n-1\right)*k_{f}-\left(n-1\right)*\phi 1*\left(k_{f}-k_{\text{s}1}\right)}{k_{\text{s}1}+\left(n-1\right)*k_{f}+\phi 1*\left(k_{f}-k_{\text{s}1}\right)}\right)$ (thermal conductivity),

$\frac{\sigma_{nf}}{\sigma_{f}}=\frac{\left(\sigma_{\text{s}1}+2*\sigma_{f}-2*\phi 1*\left(\sigma_{f}-\sigma_{\text{s}1}\right)\right)}{\left(\sigma_{\text{s}1}+2*\sigma_{f}+\phi 1*\left(\sigma_{f}-\sigma_{\text{s}1}\right)\right)}$ (electrical conductivity), And for hybrid nanofluids, the correlations are defined in the following expressions:

$\rho_{hnf}=\left\{\left(1-{ф}_{2}\right)\left[\left(1-{ф}_{1}\right)\rho_{f}+{ф}_{1}\rho_{s_{1}}\right]\right\}+{ф}_{2}\rho_{s_{2}}$ (density).

${\left(\rho C_{p}\right)}_{hnf}=\left\{\left(1-{ф}_{2}\right)\left[\left(1-{ф}_{1}\right){\left(\rho C_{p}\right)}_{f}+{ф}_{1}{\left(\rho C_{p}\right)}_{s_{1}}\right]\right\}+{ф}_{2}{\left(\rho C_{p}\right)}_{s_{2}}$ (heat capacity).

${µ}_{hnf}=\frac{{µ}_{f}}{{\left(1-{ф}_{1}\right)}^{2.5}{\left(1-{ф}_{2}\right)}^{2.5}}$ (dynamic viscosity).

$\frac{k_{hnf}}{k_{nf}}=\left(\frac{k_{\text{s}2}+\left(n-1\right)*k_{\text{nf}}-\left(n-1\right)*\phi 2*\left(k_{\text{nf}}-k_{\text{s}2}\right)}{k_{\text{s}2}+\left(n-1\right)*k_{\text{nf}}+\phi 2*\left(k_{\text{nf}}-k_{\text{s}2}\right)}\right)$ (thermal conductivity)

And $\frac{k_{nf}}{k_{f}}=\left(\frac{k_{\text{s}1}+\left(n-1\right)*k_{f}-\left(n-1\right)*\phi 1*\left(k_{f}-k_{\text{s}1}\right)}{k_{\text{s}1}+\left(n-1\right)*k_{f}+\phi 1*\left(k_{f}-k_{\text{s}1}\right)}\right)$ ,

Electrical conductivity $\frac{\sigma_{\text{hnf}}}{\sigma_{nf}}=\left(\frac{\left(\sigma_{\text{s}2}+2*\sigma_{\text{nf}}-2*\phi 2*\left(\sigma_{\text{nf}}-\sigma_{\text{s}2}\right)\right)}{\left(\sigma_{\text{s}2}+2*\sigma_{\text{nf}}+\phi 2*\left(\sigma_{\text{nf}}-\sigma_{\text{s}2}\right)\right)}\right)$ where, $\frac{\sigma_{nf}}{\sigma_{f}}=\frac{\left(\sigma_{\text{s}1}+2*\sigma_{f}-2*\phi 1*\left(\sigma_{f}-\sigma_{\text{s}1}\right)\right)}{\left(\sigma_{\text{s}1}+2*\sigma_{f}+\phi 1*\left(\sigma_{f}-\sigma_{\text{s}1}\right)\right)}$ .

In thermal conductivity correlations, n is the nanoparticle shape factor which is equal to 16.1576 (lamina shape). The specific thermophysical attribute values for the nanoparticles and hosting liquid (blood) are described in Table 1 (Ahmed et al., 2018; Hosseinzadeh et al., 2021; Ashraf et al., 2022).

**TABLE 1:** The values for thermophysical attributes and nanoparticle shape factors.

Characteristics	Density $\left(\frac{\mathrm{\rho g}}{m^{3}}\right)$	Heat capacity $\left(\frac{J}{\mathrm{Kg K}}\right)$	Thermal conductivity (W/mk)	Electrical conductivity (S/m)
Blood	1063	3594	0.492	4.3 × 10^-3^
Al_2_O_3_	3970	765	40	35 × 10^6^
Fe_3_O_4_	5180	670	9.7	6.9 × 10^-2^
Shape factor values				
Particle’s name	Shape	Shape factor vale (n)		
Lamina	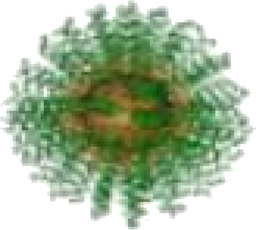	16.1576		
Platelets	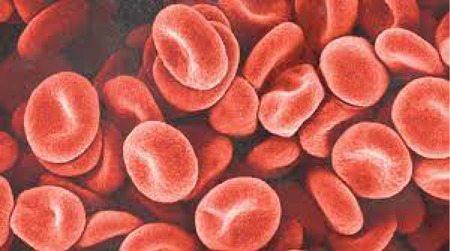	5.7		
Hexahedron	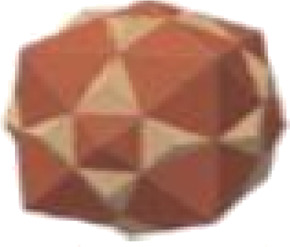	3.7221		

### Similarity rules

The following similarity equations are designated to perform the dimensional analysis of the model:

$$\{\begin{array}{c} \begin{array}{cc} \bar{u}=axF\prime^{\left(\eta \right)} & \bar{v}=ayG\prime^{\left(\eta \right)} \end{array}\\ \bar{w}=-\sqrt{av_{f}}\left[F\left(\eta \right)+G\left(\eta \right)\right]\\ \begin{array}{cc} \eta =z\sqrt{\frac{a}{v_{f}}} & \beta \left(\eta \right)=\frac{T-T_{\infty }}{T_{f}-T_{\infty }} \end{array} \end{array}$$ (6)

And, the continuity in Eq. 1 is clearly satisfied in the view of aforementioned similarity equations.

### Final (Al_2_O_3_-Fe_3_O_4_)/blood hybrid model

Using similarity transformation and thermo-physical characteristics in the governing model, the final version of the model is achieved:

$$F^{\prime \prime \prime }-{\left(1-\phi_{1}\right)}^{2.5}\left(1-\phi_{2}\right)\left\{\left(1-\phi_{2}\right)\left[\left(1-\phi_{1}\right)+\phi_{1}\left(\frac{\rho_{s_{1}}}{\rho_{f}}\right)\right]+\phi_{2}\left(\frac{\rho_{s_{2}}}{\rho_{f}}\right)\right\}\left[{\left(F^{\prime }\right)}^{2}-\left(G+F\right)F^{\prime \prime }\right]-{\left(1-\phi_{1}\right)}^{2.5}\left(1-\phi_{2}\right)M^{2}\sigma_{hnf}F^{\prime }=0,$$ (7)

$$G^{\prime \prime \prime }-{\left(1-\phi_{1}\right)}^{2.5}\left(1-\phi_{2}\right)\left\{\left(1-\phi_{2}\right)\left[\left(1-\phi_{1}\right)+\phi_{1}\left(\frac{\rho_{s_{1}}}{\rho_{f}}\right)\right]+\phi_{2}\left(\frac{\rho_{s_{2}}}{\rho_{f}}\right)\right\}\left[{\left(G^{\prime }\right)}^{2}-\left(G+F\right)G^{\prime \prime }\right]-{\left(1-\phi_{1}\right)}^{2.5}\left(1-\phi_{2}\right)M^{2}\sigma_{hnf}G^{\prime }=0,$$ (8)

$$\beta^{\prime \prime }\left(1+\frac{Rd}{\frac{k_{hnf}}{k_{f}}}\right)+\frac{Pr}{\frac{k_{hnf}}{k_{f}}}\left\{\left(1-{ф}_{2}\right)\left[\left(1-{ф}_{1}\right)+{ф}_{1}\frac{{\left(\rho C_{p}\right)}_{s_{1}}}{{\left(\rho C_{p}\right)}_{f}}\right]+\frac{{ф}_{2}{\left(\rho C_{p}\right)}_{s_{2}}}{{\left(\rho C_{p}\right)}_{f}}\right\}\left[G+F\right]\beta^{\prime }=0,$$ (9)

$$\frac{k_{hnf}}{k_{f}}=\left(\frac{k_{s2}+\left(n-1\right)*k_{\text{n}\text{f}}-\left(n-1\right)*\phi 2*\left(k_{\text{n}\text{f}}-k_{s2}\right)}{k_{s2}+\left(n-1\right)*k_{\text{n}\text{f}}+\phi 2*\left(k_{\text{n}\text{f}}-k_{s2}\right)}\right)*\left(\frac{k_{s1}+\left(n-1\right)*k_{f}-\left(n-1\right)*\phi 1*\left(k_{f}-k_{s1}\right)}{k_{s1}+\left(n-1\right)*k_{\text{n}\text{f}}\mathrm{+\phi}1*\left(k_{f}-k_{s1}\right)}\right),$$

$$\sigma_{hnf}=\left(\frac{\left(\sigma_{s2}+2*\sigma_{\text{n}\text{f}}-2*\phi 2*\left(\sigma_{\text{n}\text{f}}-\sigma_{s2}\right)\right)}{\left(\sigma_{s2}+2*\sigma_{\text{n}\text{f}}\mathrm{+\phi}2*\left(\sigma_{\text{n}\text{f}}-\sigma_{s2}\right)\right)}\right)*\left(\frac{\left(\sigma_{s1}+2*\sigma_{f}-2*\phi 1*\left(\sigma_{f}-\sigma_{s1}\right)\right)}{\left(\sigma_{s1}+2*\sigma_{f}\mathrm{+\phi}1*\left(\sigma_{f}-\sigma_{s1}\right)\right)}\right)$$

With the boundary conditions,

$$\{\begin{array}{c} \begin{array}{ccc} F^{\prime }\left(0\right)=1+\gamma_{1}F^{\prime \prime }\left(0\right), & G^{\prime }\left(0\right)=S_{t}+\gamma_{1}G^{\prime \prime }\left(0\right), & \left[F\left(0\right)+G\left(0\right)\right]\mathrm{=0,} \end{array}\\ \beta \left(0\right)=1,\\ \begin{array}{ccc} F^{\prime }\left(\infty \right)\to \mathrm{0,} & G^{\prime }\left(\infty \right)\to \mathrm{0,} & \beta \left(\infty \right)\to \mathrm{0,} \end{array} \end{array}$$ (10)

Furthermore, $S_{t}=b/a$ (stretching parameter), $M^{2}=\sigma B_{о}^{2}/a\rho_{f}$ is the magnetic interaction parameter, $Rd=16\sigma^{*}T_{\infty }^{3}/3k^{*}k_{f\;}$ is the Radiation parameter, and $\gamma_{1}=\frac{\left(2-\sigma_{v}\right)}{\sigma_{v}}\lambda_{о}\;\sqrt{a\nu_{f}^{-1}\lambda_{0}}$ (slip parameter). The skin-friction coefficient $C_{f\;},\;$ and reduced Nusselt number $Nu_{x},\;$ are defined as:

$C_{f}=\frac{{µ}_{hnf}{\left(\partial u/\partial z\right)}_{z=0}}{\rho_{f}u_{w}^{2}}$ and $Nu_{x}=-\frac{xk_{hnf}{\left(\partial T/\partial z\right)}_{z=0}}{k_{f}\left(T_{f}-T_{\infty }\right)}$ , respectively.

## Mathematical analysis

The problem in hand is solved numerically due to the high strength of the non-linear terms and it is followed by the following steps:

• Firstly, write the model in its appropriate form.
• Make substitutions according to the order of model.
• Using those substitutions, the higher-order model should be transformed into first order IVP.
• Adjust the BCs accordingly and set those conditions equal to unknowns which will be determined latter.
• Finally, run the code and plot the results for various physical constraints.

**FIGURE 2:**
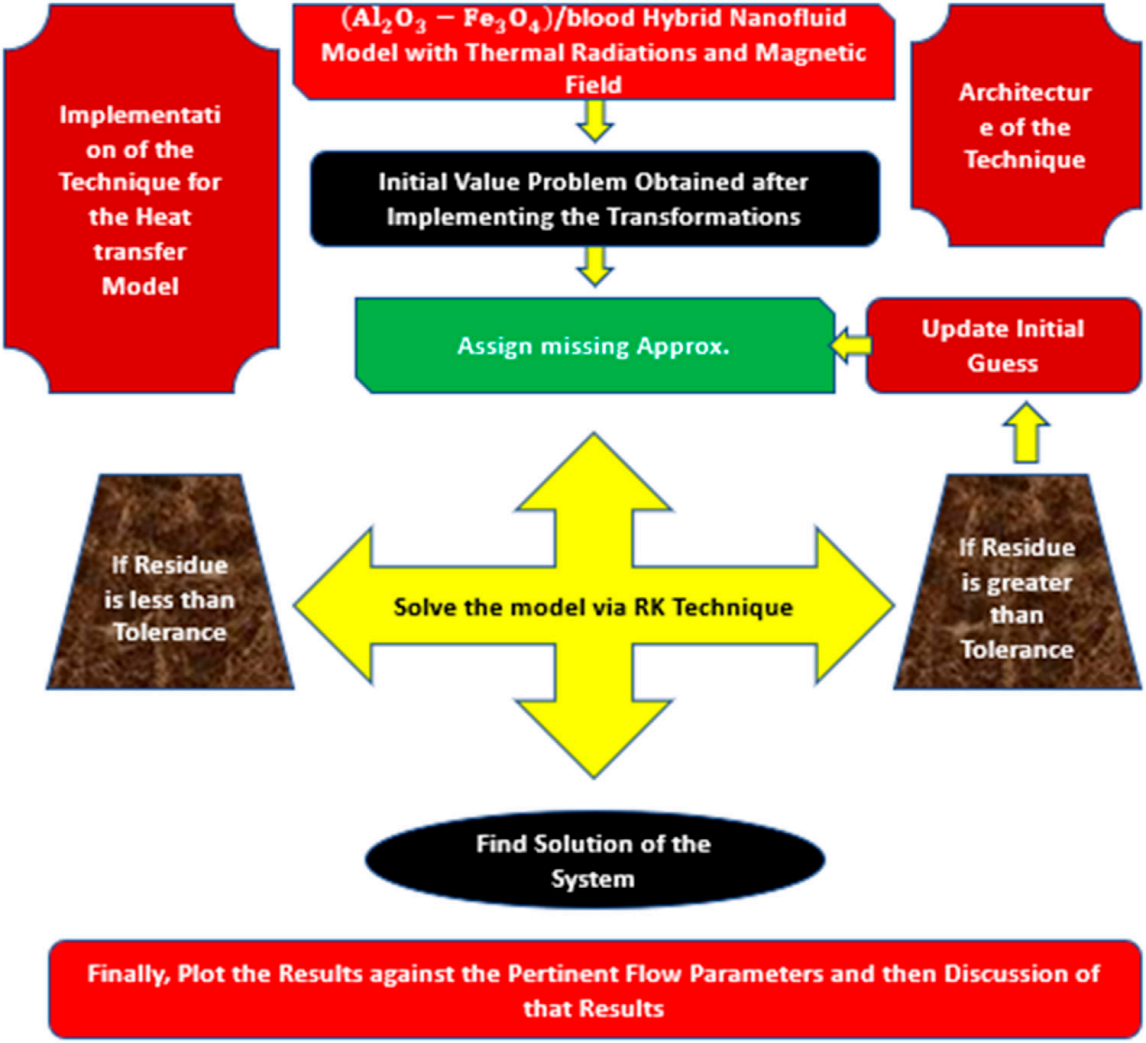
Implementation process of the technique.

The complete working rules for Runge-Kutta scheme are given in Figure 2.

By following the flow chart, the model (Al_2_O_3_-Fe_3_O_4_)/blood adjust in the following pattern:

$$F^{\prime \prime \prime }={\left(1-{ф}_{1}\right)}^{2.5}\left(1-{ф}_{2}\right)\left\{\left(1-{ф}_{2}\right)\left[\left(1-{ф}_{1}\right)+{ф}_{1}\left(\frac{\rho_{s_{1}}}{\rho_{f}}\right)\right]+{ф}_{2}\left(\frac{\rho_{s_{2}}}{\rho_{f}}\right)\right\}\left[{\left(F^{\prime }\right)}^{2}-\left(G+F\right)F^{\prime \prime }\right]+{\left(1-{ф}_{1}\right)}^{2.5}\left(1-{ф}_{2}\right)M^{2}\sigma_{hnf}F^{\prime }$$ (11)

$$G^{\prime \prime \prime }={\left(1-\phi_{1}\right)}^{2.5}\left(1-\phi_{2}\right)\left\{\left(1-\phi_{2}\right)\left[\left(1-\phi_{1}\right)+\phi_{1}\left(\frac{\rho_{s_{1}}}{\rho_{f}}\right)\right]+\phi_{2}\left(\frac{\rho_{s_{2}}}{\rho_{f}}\right)\right\}\left[{\left(G^{\prime }\right)}^{2}-\left(G+F\right)G^{\prime \prime }\right]+{\left(1-\phi_{1}\right)}^{2.5}\left(1-\phi_{2}\right)M^{2}\sigma_{hnf}G^{\prime },$$ (12)

$$\beta^{\prime \prime }=\frac{1}{\left(1+\frac{Rd}{\frac{k_{hnf}}{k_{f}}}\right)}\left[-\frac{Pr}{\frac{k_{hnf}}{k_{f}}}\left\{\left(1-{ф}_{2}\right)\left[\left(1-{ф}_{1}\right)+{ф}_{1}\frac{{\left(\rho C_{p}\right)}_{s_{1}}}{{\left(\rho C_{p}\right)}_{f}}\right]+\frac{{ф}_{2}{\left(\rho C_{p}\right)}_{s_{2}}}{{\left(\rho C_{p}\right)}_{f}}\right\}\left[G+F\right]\beta^{\prime }\right]$$ (13)

Now according to the model, the following are appropriate transformations:

$${\left[{\tilde{ℸ}}_{1}\;\;\;{\tilde{ℸ}}_{2}\;\;\;{\tilde{ℸ}}_{3}\;\;\;\tilde{ℸ}\prime {\;}_{3}\right]}^{t}={\left[F\;\;\;F^{\prime }\;\;F^{\prime \prime }\;\;F^{\prime \prime \prime }\right]}^{t}$$

$${\left[{\tilde{ℸ}}_{4}\;\;\;{\tilde{ℸ}}_{5}\;\;\;{\tilde{ℸ}}_{6}\;\;\;\tilde{ℸ}\prime {\;}_{6}\right]}^{t}={\left[G\;\;\;G^{\prime }\;\;G^{\prime \prime }\;\;G^{\prime \prime \prime }\right]}^{t}$$

$${\left[{\tilde{ℸ}}_{7}\;\;\;{\tilde{ℸ}}_{8}\;\;\;\tilde{ℸ}\prime {\;}_{8}\right]}^{t}={\left[\beta \;\;\;\beta^{\prime }\;\;\beta^{\prime \prime }\right]}^{t}$$

Eqs 11–13 are then combined in the following form:

$${\tilde{ℸ}}_{3}={\left(1-\phi_{1}\right)}^{2.5}\left(1-\phi_{2}\right)\left\{\left(1-\phi_{2}\right)\left[\left(1-\phi_{1}\right)+\phi_{1}\left(\frac{\rho_{s1}}{\rho_{f}}\right)\right]+\phi_{2}\left(\frac{\rho_{s2}}{\rho_{f}}\right)\right\}\left[{\left({\tilde{ℸ}}_{2}\right)}^{2}-\left({\tilde{ℸ}}_{1}+{\tilde{ℸ}}_{4}\right){\tilde{ℸ}}_{3}\right]+{\left(1-\phi_{1}\right)}^{2.5}\left(1-\phi_{2}\right)M^{2}{\tilde{ℸ}}_{2}\sigma_{hnf},$$

$${\tilde{ℸ}}_{6}={\left(1-\phi_{1}\right)}^{2.5}\left(1-\phi_{2}\right)\left\{\left(1-\phi_{2}\right)\left[\left(1-\phi_{1}\right)+\phi_{1}\left(\frac{\rho_{s1}}{\rho_{f}}\right)\right]+\phi_{2}\left(\frac{\rho_{s2}}{\rho_{f}}\right)\right\}\left[{\left({\tilde{ℸ}}_{5}\right)}^{2}-\left({\tilde{ℸ}}_{1}+{\tilde{ℸ}}_{4}\right){\tilde{ℸ}}_{6}\right]+{\left(1-\phi_{1}\right)}^{2.5}\left(1-\phi_{2}\right)M^{2}{\tilde{ℸ}}_{5}\sigma_{hnf},$$

$${\tilde{ℸ}}_{8}=\frac{1}{\left(\mathrm{1+}\frac{\mathrm{Rd}}{\frac{k_{\mathrm{hnf}}}{k_{f}}}\right)}\left[-\frac{\mathrm{Pr}}{\frac{k_{\mathrm{hnf}}}{k_{f}}}\left\{\left(1-\phi_{2}\right)\left[\left(1-\phi_{1}\right)+\phi_{1}\frac{{\left(\rho C_{p}\right)}_{s1}}{{\left(\rho C_{p}\right)}_{f}}\right]+\frac{\phi_{2}{\left(\rho C_{p}\right)}_{s2}}{{\left(\rho C_{p}\right)}_{f}}\right\}-\left({\tilde{ℸ}}_{1}+{\tilde{ℸ}}_{4}\right){\tilde{ℸ}}_{8}\right]$$

After this, numerical computation is performed and furnished for the results for various physical constraints over the desired region.

## Results and discussion against the physical constraints

The physical flow constraints are imperative to analyze the fluid motion and thermal behavior over a desired region. For the sake of this purpose, the results are organized to examine the hybrid nanofluid dynamics.

### The velocity behavior of (Al_2_O_3_-Fe_3_O_4_)/blood and Al_2_O_3_/blood

**FIGURE 3:**
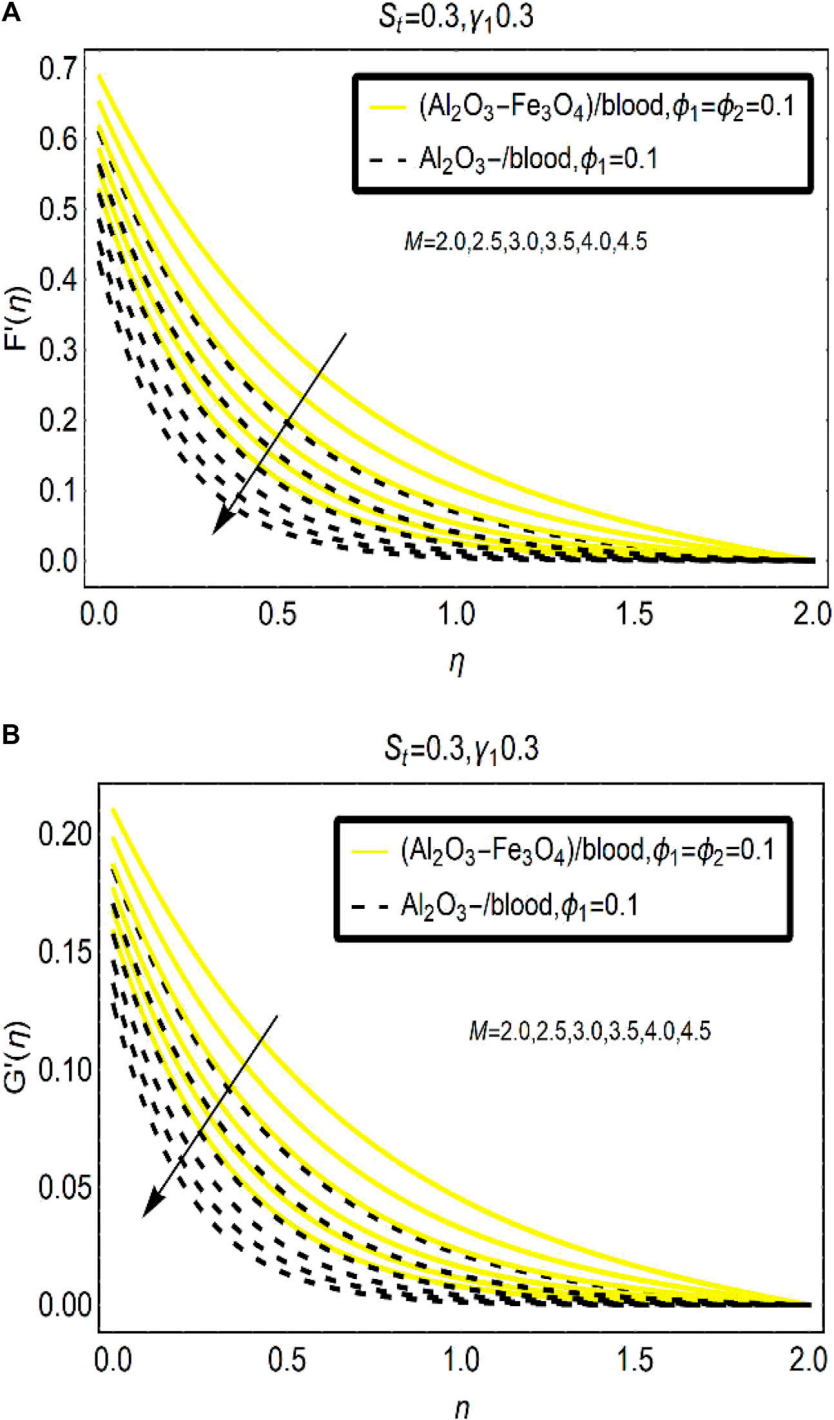
The velocity changes under varying M for **(A)** $F^{\prime }$ and **(B)** $G^{\prime }$

Figure 3 demonstrates the velocity of [(Al_2_O_3_-Fe_3_O_4_)/blood]_hnf_ and [(Al_2_O_3_)/blood]_nf_ against the imposed magnetic field (Figure 3) aligned vertically to the plane of flow. The results reveal that the velocity ( $F^{\prime }\left(\eta \right)$ and $G^{\prime }\left(\eta \right)$ ) of [(Al_2_O_3_-Fe_3_O_4_)/blood]_hnf_ and [(Al_2_O_3_)/blood]_nf_ drops for the magnetic parameter effects M. Physically, the aligned magnetic field opposes the fluid motion due to which the fluid particles move slowly. In the surroundings of the surface, these effects are optimum and the motion gradually reduces far from the sheet and finally vanishes at an ambient location from the surface. Thus, the motions of [(Al_2_O_3_-Fe_3_O_4_)/blood]_hnf_ and [(Al_2_O_3_)/blood]_nf_ can be controlled by strengthening the aligned magnetic field which is a significant physical phenomena.

**FIGURE 4:**
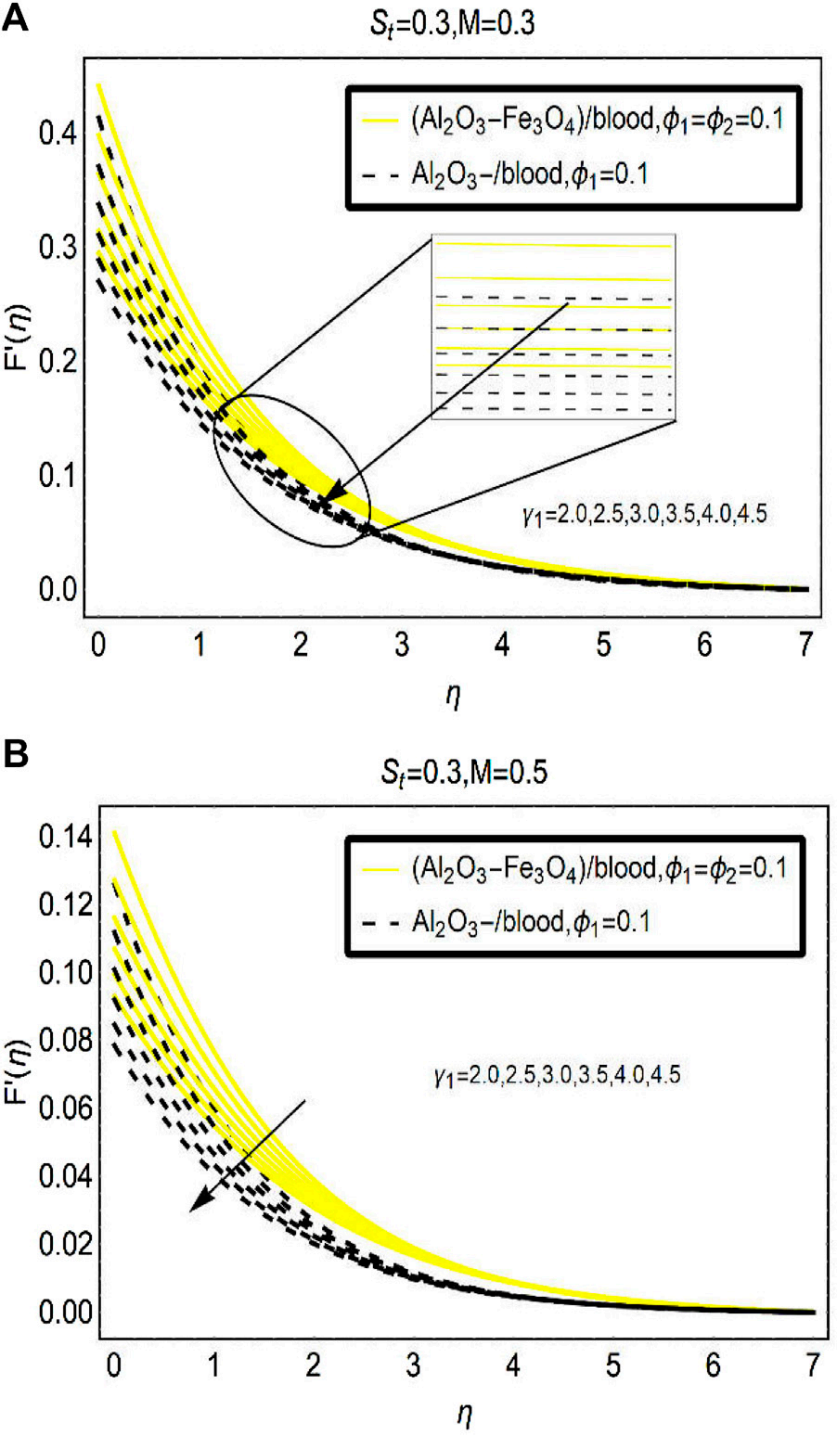
The velocity changes under varying $\gamma_{1}$ (slip parameter) for **(A)** $F^{\prime }$ and **(B)** $G^{\prime }$

The results for $F^{\prime }\left(\eta \right)$ and $G^{\prime }\left(\eta \right)$ due to a modified slip condition (slip parameter $\gamma_{1}$ ) at the surface are elaborated in Figure 4. Physically, due to the considered slip effects on the surface, the frictional force reduces between the surface and the immediate fluid layer and in the meanwhile, intermolecular forces playing the role and the fluid movement drops (the densities of the fluid improve due to mono and hybrid nanoparticles). These effects are optimum at the surface due the dominant role of the velocity slip condition and progressively decline at an ambient position. For [(Al_2_O_3_-Fe_3_O_4_)/blood]_hnf_, the motion reduces abruptly due to the higher density of the hybrid nanoparticle (Al_2_O_3_-Fe_3_O_4_). Another physical aspect of this decreasing behavior is the implementation of a magnetic field over the surface.

**FIGURE 5:**
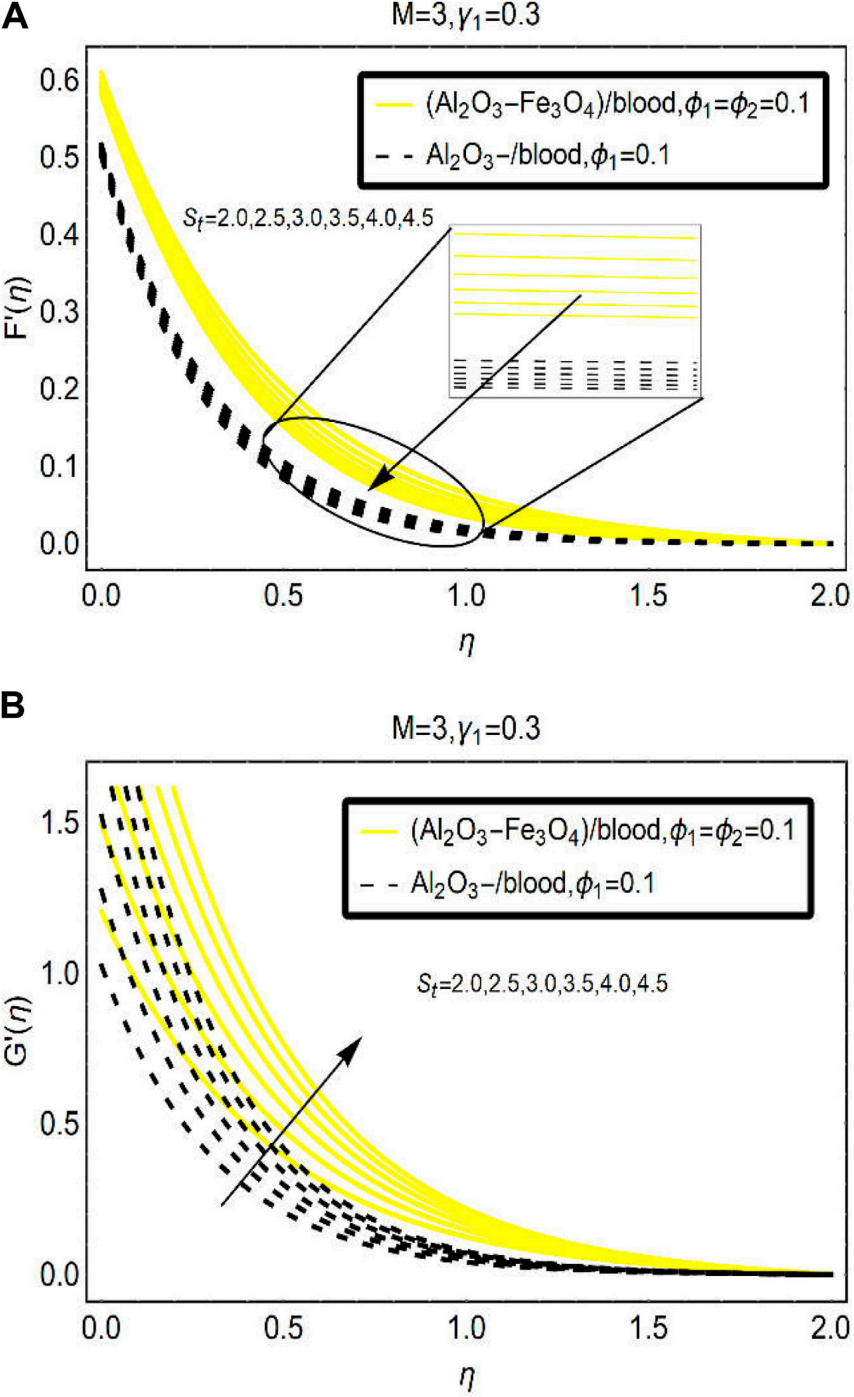
The velocity changes under varying $S_{t}$ (stretching parameter) for **(A)** $F^{\prime }$ and **(B)** $G^{\prime }$

Stretching of the surface is another physical aspect to observe the fluid movement over the region. Therefore, Figure 5 is furnished to examine the behavior of [(Al_2_O_3_-Fe_3_O_4_)/blood]_hnf_ and [(Al_2_O_3_)/blood]_nf_. Very fascinating changes in the fluid movement are observed due to stretching of the surface. It is explored that the velocity $F^{\prime }\left(\eta \right)$ reduces by increasing the parameter $S_{t}$ and an almost inconsequential movement of [(Al_2_O_3_-Fe_3_O_4_)/blood]_hnf_ and [(Al_2_O_3_)/blood]_nf_ is noticed. However, a significant increment is observed in the velocity $G^{\prime }\left(\eta \right)$ in the surrounding of the sheet. Physically, stretching of the surface enlarges the flowing region over the surface and the [(Al_2_O_3_-Fe_3_O_4_)/blood]_hnf_ and [(Al_2_O_3_)/blood]_nf_ particles freely flow over the surface due to which the momentum rises and hence the velocity significantly grows in this region.

### Thermal behavior of (Al_2_O_3_-Fe_3_O_4_)/blood and Al_2_O_3_/blood

**FIGURE 6:**
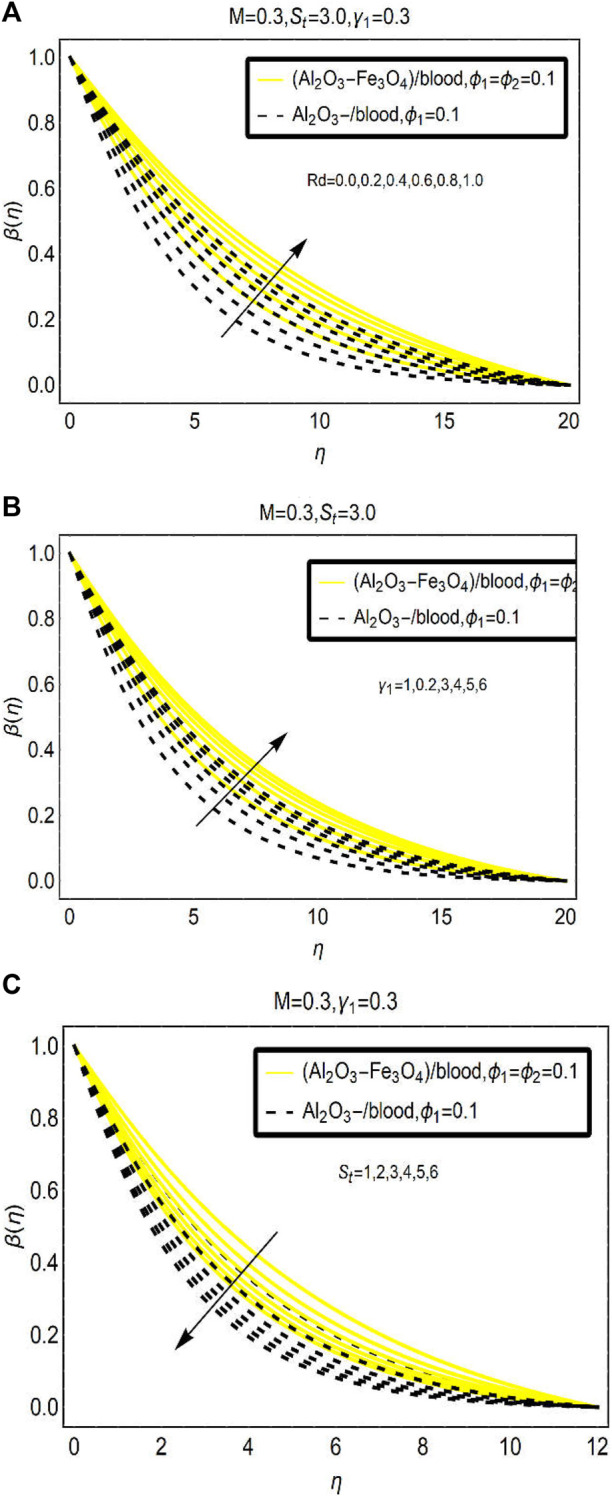
The temperature changes under varying **(A)** Rd **(B)** $\gamma_{1}$ (slip parameter), and **(C)** $S_{t}$ (stretching parameter).

The analysis of thermal enhancement in [(Al_2_O_3_-Fe_3_O_4_)/blood]_hnf_ and [(Al_2_O_3_)/blood]_nf_ is the heart of the study while dealing with nano and hybrid nanofluids. Therefore, a subsequent discussion is about the thermal enhancement in under consideration nanofluid with varying attributes of Rd, $\gamma_{1}$ , and $S_{t}$ . For this, Figure 6 is organized.

From Figure 6A, it is analyzed that applied thermal radiations over the flow configuration is a very important parameter that significantly alters the temperature characteristics of [(Al_2_O_3_-Fe_3_O_4_)/blood]_hnf_ and [(Al_2_O_3_)/blood]_nf_ over a stretching radiated surface. Figure 6A discloses that the temperature increases by strengthening the applied thermal radiations (Rd). In [(Al_2_O_3_-Fe_3_O_4_)/blood]_hnf,_ the temperature intensifies rapidly than conventional nanoliquid [(Al_2_O_3_)/blood]_nf_. Physically, thermal radiations and thermal conductivities of bi and mono nanoparticles in the base solvent (blood) improve thermal storage of the fluid. Therefore, the temperature enhances in both the nanoliquids. In a hybrid nanoliquid, the temperature changes are observed more rapidly than mono nanoliquid due to the difference between their thermal conductivities.

Similarly, Figure 6B and Figure 6C demonstrate the temperature alterations for $\gamma_{1}$ (due to slip BCs) and $S_{t}$ (due to stretching of the surface), respectively. The implementation of the slip condition became useful for thermal enhancement due to rapid collisions between the fluid particles; whereas, opposing temperature effects are examined in Figure 6C for growing values of surface-stretching parameter $S_{t}$ .

### Quantities of practical interest and thermophysical attributes

**FIGURE 7:**
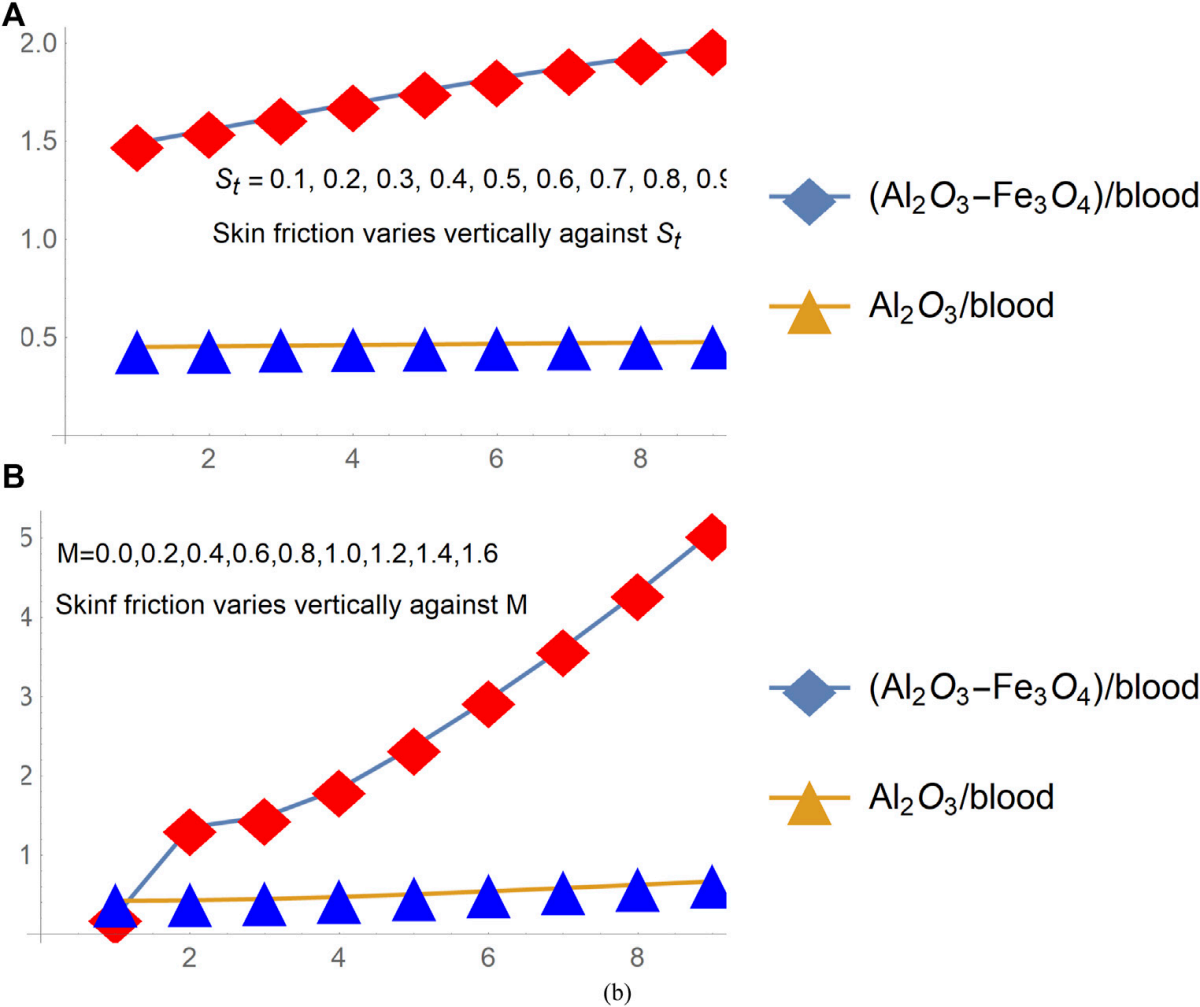
Skin friction changes under varying **(A)** $S_{t}$ (stretching parameter) and **(B)** M.

**FIGURE 8:**
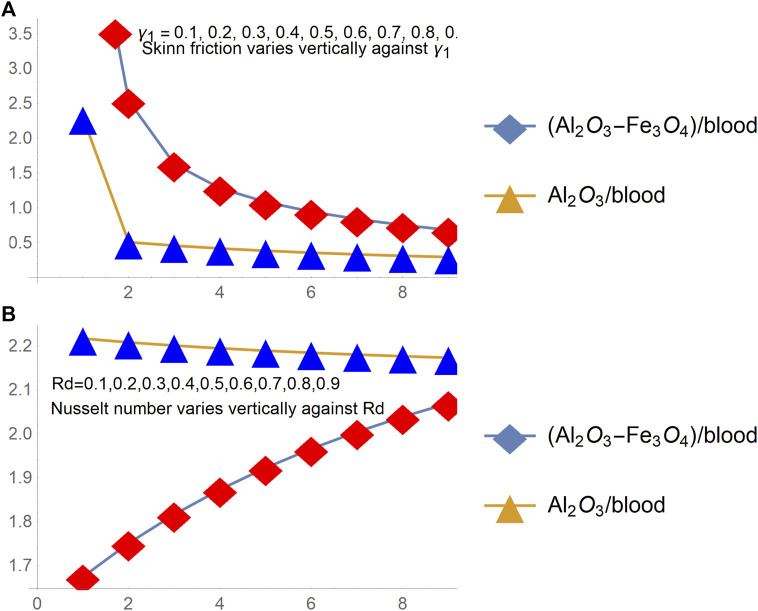
Skin friction changes under varying **(A)** $\gamma_{1}$ (slip parameter), **(B)** local Nusselt number Rd.

The study of skin friction and local heat transport rate achieved much attention of the researchers, and more specifically, engineers because of their significant contribution in various engineering applications. Thus, the behavior of shear stresses and local heat transport rate for [(Al_2_O_3_-Fe_3_O_4_)/blood]_hnf_ and [(Al_2_O_3_)/blood]_nf_ over a radiated and slippery stretchable surface is pictured in Figures 7, 8, respectively. The parameters of interest in the particular model are the stretching surface ratio ( $S_{t}$ ), magnetic number (M), slippery effects ( $\gamma_{1}$ ), and radiation number (Rd).

**FIGURE 9:**
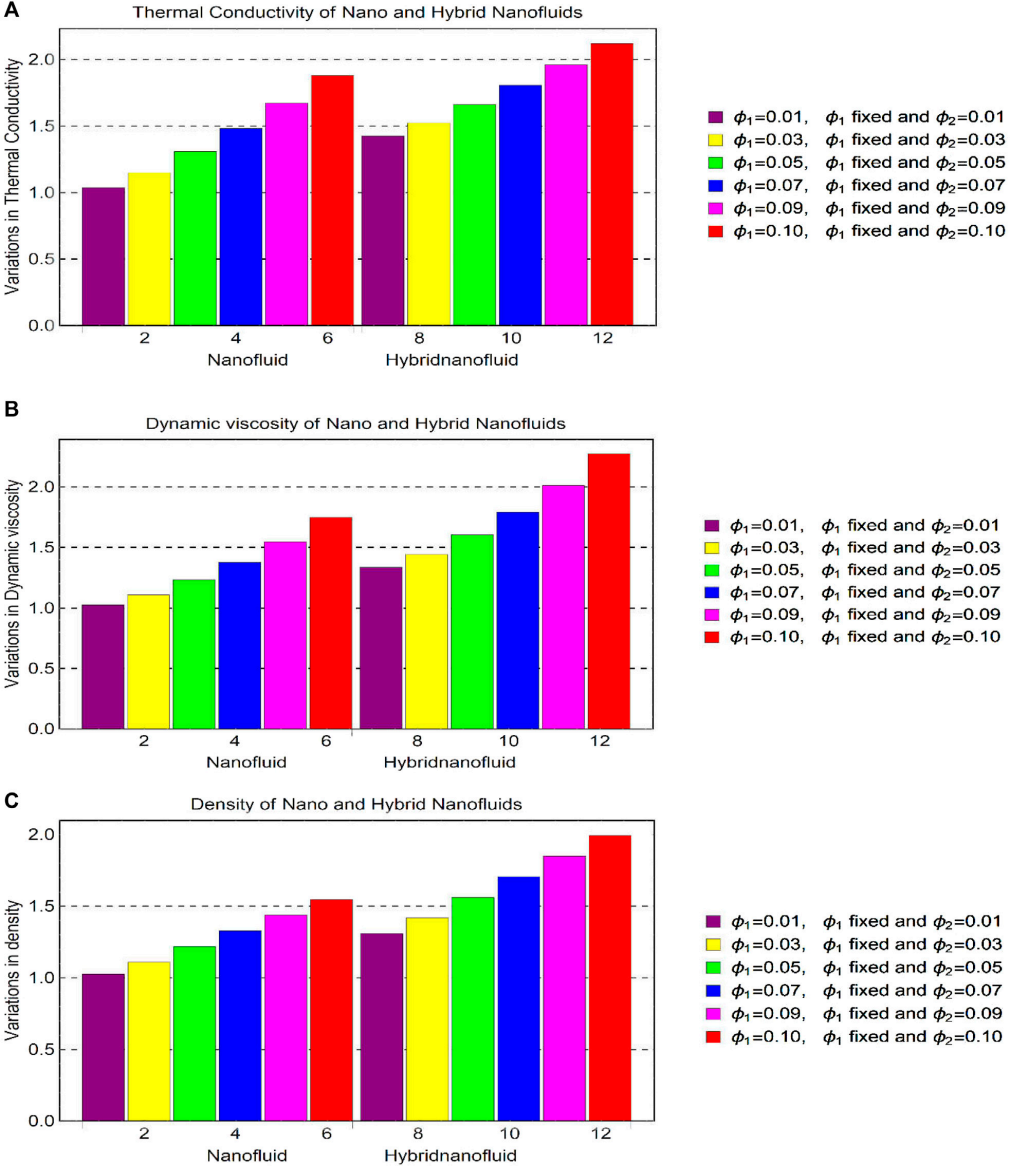
The changes in thermophysical values for **(A)** thermal conductivity **(B)** dynamic viscosity, and **(C)** density.

The analysis of Figure 8 ensures that the shear drag at the slippery surface upturns for a more stretchable and magnetized surface. The rapid growth of shear stresses is inspected for hybrid nanofluid [(Al_2_O_3_-Fe_3_O_4_)/blood]_hnf_ than regular fluid [(Al_2_O_3_)/blood]_nf_. Being a denser solution, hybrid nanofluid has this characteristic whereas; shear stress decays for growing slippery effects and these are elaborated in Figure 9A. The behavior of the local heat transport rate due to imposed thermal radiation (Rd) is furnished in Figure 9B for both [(Al_2_O_3_-Fe_3_O_4_)/blood]_hnf_ and [(Al_2_O_3_)/blood]_nf_. The results expose that induction of the thermal radiation in the constitutive model is an important physical aspect to enhance the heat transport rate in nanofluids.

Thermophysical attributes of [(Al_2_O_3_-Fe_3_O_4_)/blood]_hnf_ and [(Al_2_O_3_)/blood]_nf_ knowingly depend on the thermophysical empirical correlations. Therefore, the behavior of these quantities due to volume fraction is depicted in Figure 9 for thermal conductance, dynamic viscosity, and density. It is inspected that, by the strengthen volume fraction within a reasonable domain, the thermophysical attributes increase which lead to a significant contribution in the nano and hybrid nanoliquids. It is also evident that, due to high thermal conductance of the hybrid nanoparticles, hybrid nanoliquid has much greater ability to store thermal energy.

### Code and study of validation

**FIGURE 10:**
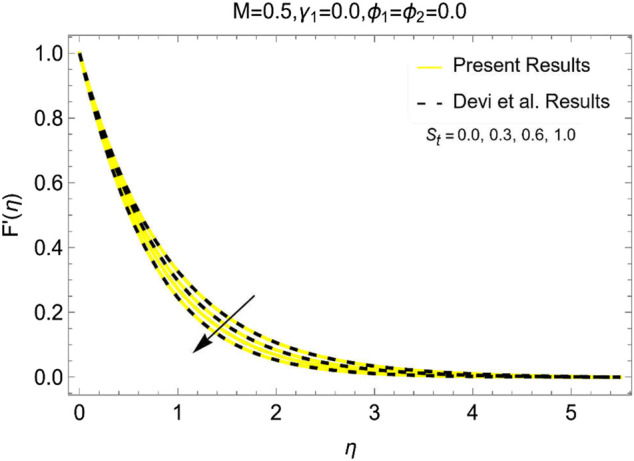
Reliability of the present analysis with previous studies.

The code and study validation with previously published data is an important factor in numerical investigation. Thus, the results of the model and code are validated with the data of Devi et al. (Devi and Devi, 2016a) by restricting the present model to some flow parameters. The comparative results for altering the stretching parameter (S_t_ = 0.0, 0.3, 0.6, 1.0) and fixed magnetic strength in Figure 10 are the evidence that the developed code and the results are valid and these can be replicated in the future.

## Conclusion

The analysis of [(Al_2_O_3_-Fe_3_O_4_)/blood]_hnf_ and [(Al_2_O_3_)/blood]_nf_ over a 3D extendable surface is conducted. The heat transport problem formulation is carried out with proper utilization of similarity equations and thermophysical models of conventional and hybrid nanofluids. Thereafter, the model is analyzed systematically through a numerical technique and the results furnished for the parameters involved determined that:

• The movement of [(Al_2_O_3_-Fe_3_O_4_)/blood]_hnf_ and [(Al_2_O_3_)/blood]_nf_ could be controlled against the high strength of the magnetic field which is beneficial for industrial applications.
• The stretching parameter is useful for rapid movement of the nanofluids over a 3D surface.
• The heat transmission ability of [(Al_2_O_3_-Fe_3_O_4_)/blood]_hnf_ due to the radiated surface is much higher than [(Al_2_O_3_)/blood]_nf._
• The % volume concentration is a core factor in hybrid and common liquids particularly in the heat storage ability.
• The skin friction and Nusselt number upsurge by intensifying the strength of the magnetic field and thermal radiations, respectively.
• The interaction of oxide nanomaterials with blood as presented in the study will contribute potentially in the field of medical and biomedical engineering.

## Data Availability

The raw data supporting the conclusions of this article will be made available by the authors, without undue reservation. The data will be provided upon reasonable request.
